# Reorganization of Dynamic Network in Stroke Patients and Its Potential for Predicting Motor Recovery

**DOI:** 10.1155/np/9932927

**Published:** 2024-12-31

**Authors:** Xiaomin Pang, Longquan Huang, Huahang He, Shaojun Xie, Jinfeng Huang, Xiaorong Ge, Tianqing Zheng, Liren Zhao, Ning Xu, Zhao Zhang

**Affiliations:** ^1^Department of Rehabilitation, The Fifth Affiliated hospital of Guangxi Medical University, The First People's Hospital of Nanning, Nanning, China; ^2^Department of Radiology, The Fifth Affiliated hospital of Guangxi Medical University, The First People's Hospital of Nanning, Nanning, China; ^3^Department of Neurology, The Fifth Affiliated hospital of Guangxi Medical University, The First People's Hospital of Nanning, Nanning, China

**Keywords:** dynamic network, ischemic stroke, motor recovery, multilayer network analysis, switching rate

## Abstract

**Objective:** The investigation of brain functional network dynamics offers a promising approach to understanding network reorganization poststroke. This study aims to explore the dynamic network configurations associated with motor recovery in stroke patients and assess their predictive potential using multilayer network analysis.

**Methods:** Resting-state functional magnetic resonance imaging data were collected from patients with subacute stroke within 2 weeks of onset and from matched healthy controls (HCs). Group-independent component analysis and a sliding window approach were utilized to construct dynamic functional networks. A multilayer network model was applied to quantify the switching rates of individual nodes, subnetworks, and the global network across the dynamic network. Correlation analyses assessed the relationship between switching rates and motor function recovery, while linear regression models evaluated the predictive potential of global network switching rate on motor recovery outcomes.

**Results:** Stroke patients exhibited a significant increase in the switching rates of specific brain regions, including the medial frontal gyrus, precentral gyrus, inferior parietal lobule, anterior cingulate, superior frontal gyrus, and postcentral gyrus, compared to HCs. Additionally, elevated switching rates were observed in the frontoparietal network, default mode network, cerebellar network, and in the global network. These increased switching rates were positively correlated with baseline Fugl–Meyer assessment (FMA) scores and changes in FMA scores at 90 days poststroke. Importantly, the global network's switching rate emerged as a significant predictor of motor recovery in stroke patients.

**Conclusions:** The reorganization of dynamic network configurations in stroke patients reveals crucial insights into the mechanisms of motor recovery. These findings suggest that metrics of dynamic network reorganization, particularly global network switching rate, may offer a robust predictor of motor recovery.

## 1. Introduction

Ischemic stroke is a prevalent condition that frequently results in deficits in cognition, language, sensation, vision, motor function, and other neurological areas, significantly impacting activities of daily living and quality of life [1, 2]. The recovery of motor function is crucial for patients to regain independence [3]. Early prediction of motor function outcomes poststroke is vital for guiding physicians in developing suitable rehabilitation programs and aiding patients and caregivers in making informed decisions about future care and placement [4]. However, relying solely on clinical assessments, such as the Fugl–Meyer assessment (FMA) scale, for predicting motor recovery poses challenges in precision [5]. While the presence of motor-evoked potential may serve as a valuable neurophysiological biomarker, its predictive value for motor recovery and outcomes is primarily confined to the upper limb [6]. Structural neuroimaging alone falls short in predicting poststroke recovery, as it offers limited insights into the functional compensatory potential of intact brain regions [7]. The utility of diffusion tensor imaging as a predictor during the acute phase remains contentious, with evidence suggesting its limited capacity to detect corticospinal tract fiber damage within the first 2 weeks poststroke [8, 9]. Consequently, there is a notable lack of biomarkers that can accurately predict motor functional recovery in the acute or subacute phases of stroke.

Over the past decade, there has been a growing interest in resting-state functional magnetic resonance imaging (rs-fMRI) biomarkers for motor recovery following stroke. Early studies focused on the predictive ability of the laterality index of the ipsilesional primary motor cortex and the functional connectivity between the bilateral primary motor cortices [10]. The introduction of a graph-theoretic approach has further emphasized that stroke is a large-scale network disorder beyond focal lesions. Reductions in local specialization, integration capacity, small-world properties, and module interaction of the brain network have been correlated with clinical measures quantifying functional recovery, providing better explanations for the changes in brain functional reorganization during stroke recovery [11–14]. Certain topological properties, such as characteristic path length and hub disruption index, have also been described as indicators for predicting motor function recovery [15, 16]. However, these previous studies used static network analysis methods and overlooked the dynamic characteristics of networks. Indeed, functional connectivity and network organization are not static but fluctuate over short timescales, even during rs-fMRI scanning [17, 18]. Investigating dynamics of brain functional networks may offer a more effective and robust approach to unveiling network reorganization associated with stroke [19]. Previous studies have demonstrated the efficacy of dynamic network features as predictors for stroke recovery [20, 21].

Multilayer network analysis offers an innovative approach to processing the complex data of dynamic networks [22]. This methodology enables the investigation of temporal and spatial variations within network configurations facilitating the tracking and quantification of temporal changes in each node within a multilayer temporal network and their switching between different modules or network assignments over time [23]. The ability of network switching has shown significant correlations with motor learning [24], executive cognition [25], recognition memory, and strategic attention [26]. Furthermore, the network switching rate has proven to be a reliable predictor of performance in cognitively demanding behavioral tasks [23]. The application of a multilayer temporal network model has unveiled abnormal dynamic network configurations in patients with various neurological disorders, including migraine, epilepsy, depression, and mild cognitive impairment [27–30]. Nevertheless, to date, no studies have employed this methodology to examine the dynamic network abnormalities following stroke, nor have they thoroughly investigated its relationship with functional outcomes poststroke or its predictive capacity.

In this study, we collected fMRI data from patients with subacute stroke within 2 weeks of symptom onset, as well as from matched healthy controls (HCs). We employed group-independent component (IC) analysis and a sliding window method to construct dynamic networks. The multilayer network model was applied to measure the switching rate of node, subnetwork, and the global network across the dynamic network. Correlation analyses were conducted to evaluate the relationship between the switching rate and motor function recovery. Linear regression models were used to further test whether the global network switching rate could predict motor recovery in stroke patients. Our hypothesis posits that network switching rates are associated with motor recovery in stroke and could be used as potential predictors.

## 2. Materials and Methods

### 2.1. Participants

A cohort of 20 patients diagnosed with ischemic stroke was recruited from the Rehabilitation Department of the Fifth Affiliated Hospital of Guangxi Medical University between July 2022 and January 2024. Inclusion criteria for recruitment were as follows: (1) age between 18 and 75 years; (2) first occurrence of unilateral subcortical stroke; (3) moderate stroke severity, as indicated by a National Institute of Health Stroke Scale (NIHSS) score of 5–15 points; and (4) enrollment within 2 weeks of symptom onset. Exclusion criteria were as follows: (1) overall poor physical condition; (2) significant cognitive and language impairments; (3) history of mental or neurological disorders other than stroke; (4) history of substance or alcohol abuse; (5) any contraindications for MRI, such as metallic implants or the presence of a cardiac pacemaker; and (6) inability to cooperate with experimental procedures. During the same recruitment period, an additional 20 individuals without a history of mental or neurological disorders were recruited as the HC group from the same community. These controls were matched to the stroke patients by age and gender. The study protocol was approved by the Medical Research Ethics Committee of the Fifth Affiliated Hospital of the Guangxi Medical University (No. 2021-078-01), and written informed consent was obtained from all participants.

### 2.2. Assessment of Motor Impairment

The FMA was employed to evaluate motor impairment in stroke patients. This assessment is widely validated and effective in assessing movement quality and synergy within this population [31]. Scores range from 0 to 100, with higher scores indicating superior motor control. Trained rehabilitation physicians obtained FMA scores at two time points: baseline and 90 days poststroke. Motor recovery was quantified as the change in FMA score, defined as the difference between these two time points (baseline and 90 days poststroke).

### 2.3. MRI Data Acquisition

MRI data acquisition for all participants was conducted using a Discovery MR750 3.0T MRI scanner (General Electric, Milwaukee, WI, USA) equipped with a standard eight-channel head coil. Foam padding was utilized to minimize head movements, and earplugs were provided to reduce scanner noise. During scanning, participants were instructed to remain awake and relaxed, with their eyes closed, and avoid focusing on any specific thoughts. fMRI data were acquired using an echoplanar imaging sequence with the following parameters: repetition time = 2000 ms; echo time = 30 ms; flip angle = 90°; field of view = 240 mm × 240 mm; matrix = 64 × 64; slice thickness = 3.6 mm; number of slices = 39; and no slice gap. Additional MR sequences for clinical diagnosis, including T1-weighted imaging (T1WI), T2-weighted imaging (T2WI), T2 fluid-attenuated inversion recovery (FLAIR), and diffusion-weighted imaging (DWI), were also acquired but were not used in this study.

### 2.4. Preprocessing of fMRI Data

Preprocessing of fMRI data was executed using GRETNA: a graph theoretical network analysis toolbox designed for imaging connectomes [32] (http://www.nitrc.org/projects/gretna/), within the Matrix Laboratory (MATLAB R2013b, www.mathworks.com/products/matlab/) environment. DICOM data generated by the MRI scanner were converted into NIfTI format. To achieve magnetization equilibrium, the initial 10 volumes of each participant's fMRI data were discarded. The subsequent volumes underwent slice timing correction and realignment procedures. To address the issue of head motion, participants were excluded from the analysis if their mean frame-wise displacement (FD) values exceeded 0.2 mm, their maximum displacement was greater than 3 mm, or if their angular rotation surpassed 3° [33]. The functional images of each participant were normalized to the EPI template and resampled to a voxel size of 3 mm × 3 mm × 3 mm. Subsequently, the normalized images were subjected to spatial smoothing using a 4 mm full-width at half-maximum Gaussian kernel [34].

### 2.5. Group Independent Component Analysis

Following data preprocessing, spatial group Independent Component Analysis (ICA) was conducted to decompose the data into functional components using the group ICA of fMRI toolbox (GIFT v4.0b, http://icatb.sourceforge.net/). The ICA pipeline adhered to the procedures outlined in the classic article by Allen et al. [35]. A high model order (number of components, *C* = 50) was selected to achieve a “functional parcellation” of both cortical and subcortical regions. Data from all participants were concatenated into a single dataset and subsequently subjected to dimensionality reduction through two stages of principal component analysis, encompassing both subject-specific and group-level steps. The reliability and stability of the infomax ICA algorithm were ensured by repeating the Algorithm 20 times using ICASSO. Following this, the spatial maps and time courses were reconstructed retrospectively for each subject. Finally, 43 meaningful ICs were identified according to the procedure described previously [36, 37]. These ICs were grouped into nine functional networks: visual network (VIS), somatomotor network (SMN), dorsal attention network (DAN), ventral attention network (VAN), limbic network (LIM), frontoparietal network (FPN), default mode network (DMN), subcortical network (SCN), and cerebellar network (CBN) [38].

### 2.6. Network Construction

Prior to network construction, additional processing of remaining noise sources was conducted, including detrending, despiking, and filtering. The sliding window method was employed to segment the time series into 210 overlapping windows, each with a window size of 20 TRs and a step length of 1 TR [39]. The 43 ICs were designated as nodes. For each sliding window, the Pearson's correlation coefficient of the time series between each pair of nodes was computed to form the dynamic network matrices. Negative values in these matrices were set to zero. This process resulted in a multilayer temporal network comprising 210 layers and 43 × 43 correlation matrices per layer. Subsequently, a super-adjacency matrix was constructed to integrate the layers, with ordinal links added to connect a node to itself in adjacent layers [40].

### 2.7. Multilayer Network Analysis

An iterative Louvain multilayer modularity algorithm was utilized to examine the dynamic reconfiguration of the multilayer temporal network. This was executed using open-source code (https://github.com/GenLouvain/GenLouvain). The algorithm partitions the modules in a multilayer network by optimizing the multilayer modular quality factor *Q*, which ranges from 0 to 1. A higher *Q* value indicates greater network segregation. This measure is governed by the parameters *γ* and *ω*, which determine the strength of topological and temporal connectivity, respectively. We used the settings *γ* = 0.9 and *ω* = 0.75. After obtaining the multilayer module assignments, the switching rate for a node was calculated as the percentage of time frames during which the node changed to a different network assignment. Switching rates of the global network and subnetworks were determined by averaging the rates of nodes belonging to the global brain network and subnetworks. To reduce the randomness of the iterative Louvain algorithm, the multilayer community detection method was conducted 50 times for each individual, and the network metrics obtained from these 50 iterations were subsequently averaged to derive the final outcome.

### 2.8. Statistical Analysis

Statistical analyses were conducted using SPSS software (V 22.0, https://www.ibm.com/spss). The normality of quantitative data was assessed using the one-sample Kolmogorov–Smirnov test. Group comparisons were executed using independent-sample *t*-tests or Mann–Whitney *U*-tests, depending on the data distribution. Differences in categorical data were examined with chi-square tests. Correlation analyses were performed to investigate the relationship between abnormalities in switching rate and FMA scores in stroke patients. Linear regression models were developed to predict motor recovery, with the change in FMA scores as the response variable and various predictors, including baseline FMA scores and global network switching rates. Model parameters were estimated using the least squares method without regularization. The goodness-of-fit test for each model was evaluated using the coefficient of determination (*R*^2^), root-mean square error (RMSE), and Akaike information criterion (AIC). Statistical significance was determined at *p* < 0.05.

### 2.9. Validation Analysis

To ensure the robustness and reproducibility of our findings, we examined the impact of various parameter choices on our analysis results. Specifically, we tested a different number of network nodes by setting 100 components in ICA. Additionally, we altered the parameters of the sliding window, using a window size of 50 TRs with a step length of 1 TR. We also varied the parameters for the multilayer network model, adjusting *γ* to 1 and 1.1 and *ω* to 0.5 and 1.

## 3. Results

### 3.1. Demographics and Clinical Characteristics

The demographic and clinical data are presented in Table 1. No significant differences were observed between the two groups regarding age, sex, and handedness (*p* > 0.05).

### 3.2. Identification of Functional Components

**Figure 1:**
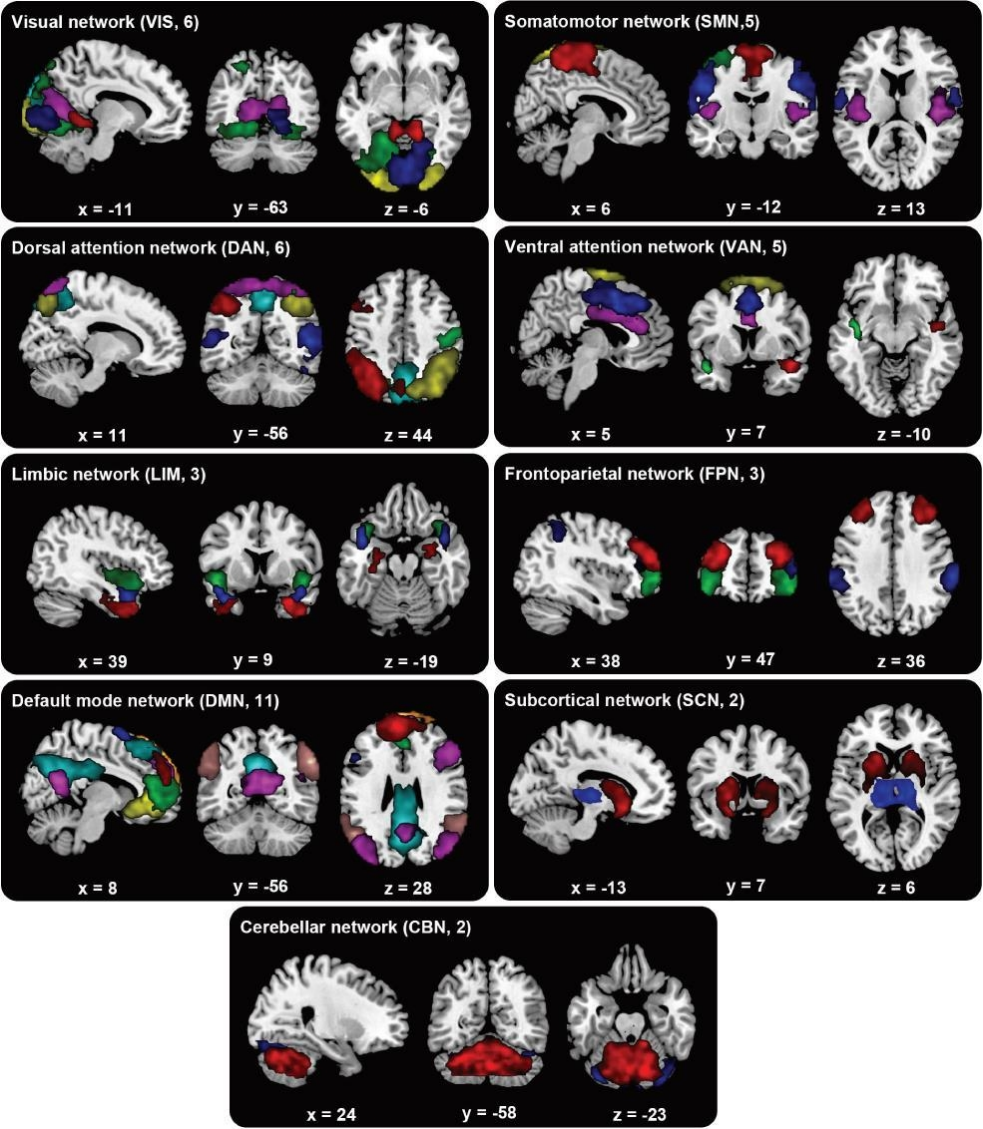
Spatial maps of the 43 meaningful ICs identified by group ICA. IC, independent component; ICA, independent component analysis.

Spatial maps of the 43 ICs are presented in Figure 1. Based on their anatomical and functional properties, these ICs were categorized into nine functional networks: VIS (IC8, IC15, IC16, IC33, IC34, IC46), SMN (IC5, IC20, IC26, IC42, IC44), DAN (IC11, IC13, IC14, IC25, IC27, IC35, IC43), VAN (IC17, IC18, IC31, IC47), LIM (IC6, IC7, IC32), FPN (IC21, IC28, IC30), DMN (IC1, IC3, IC9, IC12, IC23, IC37, IC39, IC41, IC48, IC49, IC50), SCN (IC10, IC19), and CBN (IC2, IC38). Detail coordinates for the peak activations are listed in Supporting Information: Table S1.

### 3.3. Modularity of Multilayer Network

**Figure 2:**
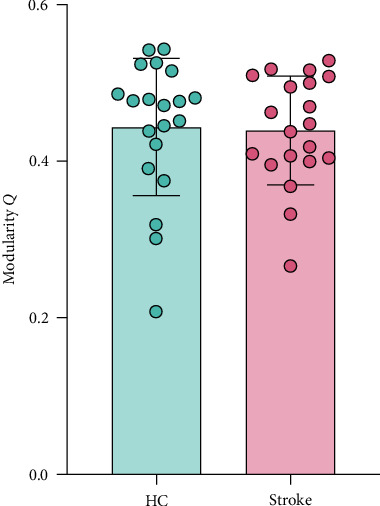
Comparison of the modularity Q of the multilayer network between two groups. HC, health control.

The quality of modularity was quantified by the modularity *Q* of the multilayer network. As shown in Figure 2, there was no significant difference between the two groups in the global modularity *Q* (*p* > 0.05).

### 3.4. Switching Rate of Node, Subnetwork, and Global Network

**Figure 3:**
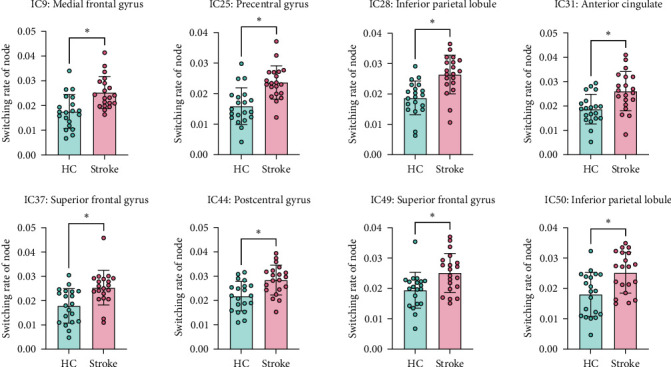
Group differences in the switching rate of nodes between two groups. IC, independent component. *⁣*^*∗*^*p* < 0.05, FDR corrected.

At the node level, the patient group exhibited a significantly increased switching rate in the IC9 (medial frontal gyrus), IC25 (precentral gyrus), IC28 (inferior parietal lobule), IC31 (anterior cingulate), IC37 (superior frontal gyrus), IC44 (postcentral gyrus), IC49 (superior frontal gyrus), and IC50 (inferior parietal lobule) compared to those in the HC group (*p* < 0.05, FDR corrected). No significant differences were detected among other nodes (*p* > 0.05, FDR corrected), as shown in Figure 3.

**Figure 4:**
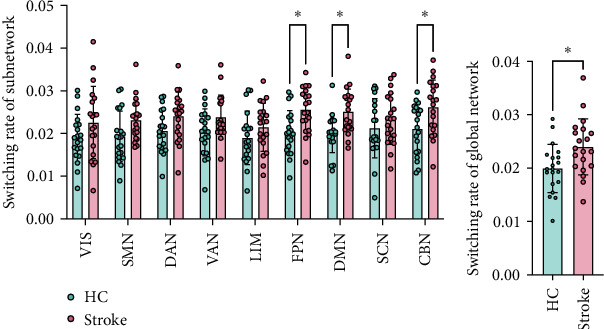
Group differences in the switching rate of subnetwork and global network between two groups. (A) *⁣*^*∗*^ Significant difference in the switching rate of subnetwork, with *p* < 0.05 after FDR correction; (B) *⁣*^*∗*^ Significant difference in the switching rate of global network, with *p* < 0.05.

At the subnetwork level, a significant increase in switching rate was observed in the FPN, DMN, and CBN of the patient group compared to the HC group (*p* < 0.05, FDR corrected). No significant differences were found in the VIS, SMN, DAN, VAN, and LIM between the groups (*p* > 0.05, FDR corrected), as shown in Figure 4A.

At the global network level, the switching rate was significantly increased in the patient group compared to the HC group (*p* < 0.05), as shown in Figure 4B.

### 3.5. Relationship Between Switching Rate and Motor Function and Its Predictive Power

**Figure 5:**
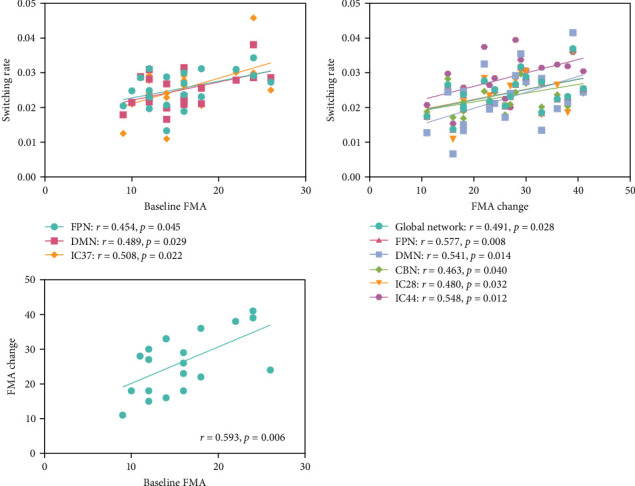
Correlation between the switching rate alterations and FMA scores in stroke patients. FMA, Fugl–Meyer assessment.

As shown in Figure 5, significant positive correlations were found between baseline FMA scores and the switching rate of IC 37 (superior frontal gyrus), as well as DMN and FPN; and between FMA change scores and IC28 (inferior parietal lobule), IC 44 (postcentral gyrus), DMN, FPN, CBN, and the global network (*p* < 0.05, uncorrected). A significant positive correlation was also observed between baseline FMA scores and FMA change scores (*p* < 0.05).

The results of the linear regression models indicated that the two regression equations were significant (*p* < 0.05), as shown in Table 2. Baseline FMA scores and global network switching rate were statistically significant predictors, with the former exhibiting higher *R*^2^ and lower RMSE and AIC values.

### 3.6. Validation

To test the reproducibility of our results, we assessed the impact of the different parameter choices on our analysis results. The main findings were largely maintained across a variety of analytical strategies (Supporting Information: Table S2).

## 4. Discussion

Stroke is increasingly acknowledged as a large-scale network disorder that extends beyond the ischemic lesion itself. In this study, we investigated the dynamic network configurations related to motor recovery in stroke patients by quantifying network switching rates. Our findings revealed a significant increase in the switching rates of specific nodes, subnetworks, and the global network in stroke patients. The increase in switching rates exhibited a positive correlation with both baseline FMA scores and changes in FMA scores at 90 days poststroke. Notably, the global network switching rate emerged as a significant predictor for motor recovery in stroke patients. These findings expand our understanding of the mechanisms underlying dynamic network reorganization following stroke and suggest that these metrics may offer robust predictors of recovery outcomes for stroke patients.

The modular architecture of brain networks facilitates efficient information exchange between different regions, enabling rapid specialization in response to varying processing demands [41–43]. Siegel et al. [44] examined the modular properties of static networks in stroke patients and found a significant reduction in brain modularity at subacute stages, with partial recovery evident at 3 months post-stroke. Their findings indicated a concurrent recovery of modular network organization and functions related to language and attention, highlighting the potential significance of normalizing large-scale modular brain systems in stroke recovery. The modular organization of brain networks is not static but reconfigures adaptively over time [24]. In this study, utilizing the multilayer networks model, we obtained more complex spatio-temporal information on dynamic network configuration. We found that the quality of the module in the multilayer network, quantified by the global modularity Q, did not show significant differences between stroke patients and HC. Given that the brain's remarkable adaptability and plasticity [45], we inferred that the functional integration within the dynamic network remains normal, potentially reflecting the brain's restructuring processes after less severe injury.

To quantify the fine-scale architectural fluctuations of modules, we measured the switching rate of each node within the multilayer network. In comparison to HC, stroke patients exhibited significantly increased switching rates of the medial frontal gyrus, precentral gyrus, inferior parietal lobule, anterior cingulate, superior frontal gyrus, and postcentral gyrus. These regions, which are primarily located in the primary motor cortex and primary somatosensory cortex, as well as in the frontoparietal cortex, are crucial for higher-order cognitive functions. Cognition-related regions are particularly involved in the early stages of motor learning poststroke [46]. Motor function recovery was correlated with activations in motor-related regions as well as several prefrontal cortex areas [47]. Preserved cognitive function significantly impacts motor recovery in stroke patients [48, 49]. The observed increase in switching rates in cognition-related regions suggests frequent interactions across the multilayer temporal network, indicating compensatory brain dynamics after stroke.

At the subnetwork level, we observed a significant increase in the switching rates in the FPN, DMN, and CBN among stroke patients. Previous studies have extensively documented alterations in connectivity patterns both within and between large-scale networks in stroke patients, encompassing not only SMN but also higher-order cognitive networks such as the FPN and DMN [50, 51]. Dysfunctions of these networks are closely linked to motor and cognitive impairments [52–55]. Longitudinal investigations have examined network reorganization following stroke, revealing a network-specific dynamic functional reorganization characterized by increased activation or interaction of the FPN and DMN during motor recovery [56, 57]. The FPN is a task-positive network primarily involved in attention-demanding cognitive tasks [51], while the DMN serves as a neurological foundation for self-referential processing and is essential for internal cognitive functions [58]. The contribution of these two cognitive networks to processes such as motor imagery, action judgments, error monitoring, and attentional control is pivotal for acquiring motor skills. They may exert a top-down influence on the motor network to regulate motor output and facilitate motor performance recovery. Consistent with these findings, our study demonstrated that increased network switching rates in the DMN and FPN are positively correlated with motor function performance. This suggests that cognition-related networks may fluctuate towards an optimal dynamic connectivity pattern to achieve functional stability or recovery following a stroke.

The cerebellum is bidirectionally connected with the neocortex via cerebello-thalamo-cortical and cortico-ponto-cerebellar pathways [59]. It plays a crucial role in sensorimotor control and motor learning, supporting plastic and adaptive cortical processes during stroke recovery [60]. Recently, the cortical CBN has been considered a potential target for interventional strategies in poststroke neuromodulation, which may provide an opportunity to achieve a more rapid and complete recovery of motor function [61–64]. The observed increase in CBN switching rate in this study provides dynamic network evidence of the involvement of the cerebellum in network configuration after stroke, supporting its key role in functional restructuring after stroke.

At the global network level, stroke patients also showed a significantly increased switching rate compared to HC. Correlation analysis revealed that the increased global network switching rate positively correlates with FMA change scores. Furthermore, the regression results indicated that the global network switching rate was a significant predictor of motor recovery in stroke patients. These findings were derived from a relatively small sample, and the conclusions must be interpreted with caution. Nevertheless, we provided an interesting perspective that measures of dynamic network reorganization could be potential biomarkers for predicting motor recovery in stroke. We plan to expand our sample size to further validate these findings in the future.

## 5. Conclusion

Our study investigated the dynamic network configuration related to motor recovery in stroke patients, revealing a significant increase in the switching rate of specific cognition-related nodes, subnetworks, and the global network. The increased switching rate was positively correlated with motor function recovery, suggesting that frequent interactions across the multilayer temporal network represent a compensatory brain dynamic mechanism following stroke. Importantly, the switching rate of the global network emerged as a significant predictor of motor recovery in stroke patients. These findings enhance our understanding of dynamic network reorganization associated with motor recovery in stroke and may offer robust predictors for patient recovery.

## Figures and Tables

**Table 1:** Demographic and clinical characteristics of patients with ischemic stroke and HC.

Characteristics	HC (*n* = 20)	Patients with ischemic stroke (*n* = 20)	Statistical value	*p* value
Age (years)	56.10 ± 8.93	59.15 ± 13.46	−0.845	0.404
Sex (male/female)	10/10	12/8	0.404	0.525
Handedness (right/left)	20/0	20/0	NA	NA
Lesion side (right/left)	NA	11/9	NA	NA
The time interval from stroke (days)	NA	12.70 ± 2.13	NA	NA
Baseline NIHSS (scores)	NA	7.00 ± 2.70	NA	NA
Baseline FMA (scores)	NA	15.80 ± 4.91	NA	NA
FMA change (scores)	NA	26.25 ± 8.68	NA	NA

Abbreviations: FMA, Fugl–Meyer assessment; HC, health control; NIHSS, National Institute of Health Stroke Scale.

**Table 2:** Results of the regression analyses.

Models	Variables	*B*	*β*	*p*	R2	RMSE	AIC
1	Baseline FMA	1.61	0.964	<0.001	0.929	7.55	81.86
2	Global network switching rate	1082.07	0.962	<0.001	0.926	7.70	82.63

Abberivations: AIC, Akaike information criterion; FMA, Fugl–Meyer assessment; RMSE, root-mean square error.

## Data Availability

The data that support the findings of this study are available from the corresponding author upon reasonable request.

## Supporting Information

Supporting Information Table S1. Peak Coordinates of functional independent components using spatial group ICA (50 ICs). Table S2. Differences in network switching rates between stroke patients and HCs compared using different parameters.
