# Supplementary material for: Reorganization of Dynamic Network in Stroke Patients and Its Potential for Predicting Motor Recovery
Source: Neural Plast. 2024 Dec 31;2024:9932927. doi: 10.1155/np/9932927 (PMC11707127; doi:10.1155/np/9932927)
Supplement: Supporting Information — Table S1. Peak Coordinates of functional independent components using spatial group ICA (50 ICs). Table S2. Differences in network switching rates between stroke patients and HCs compared using different parameters. [file 9932927.f1.docx]

**Supplementary Table 1.** Peak Coordinates of functional independent components using spatial group ICA (50 ICs).

| Components | Regions | T-value | MNI coordinate | | |
| --- | --- | --- | --- | --- | --- |
|  |  |  | x | y | z |
| Visual network (VIS) | | | | | |
| IC8 | Lingual Gyrus | 12.718 | 15 | -45 | -6 |
| IC15 | Lingual Gyrus | 18.191 | 6 | -75 | -3 |
| IC16 | Lingual Gyrus | 16.4648 | -21 | -54 | -9 |
| IC33 | Cuneus | 16.0648 | 12 | -57 | 3 |
| IC34 | Middle Occipital Gyrus | 12.9985 | -6 | -87 | -6 |
| IC46 | Cuneus | 18.0172 | 15 | -99 | 24 |
| Somatomotor network (SMN) | | | | | |
| IC5 | Postcentral Gyrus | 15.5231 | 0 | -27 | 78 |
| IC20 | Precentral Gyrus | 13.3779 | -63 | -3 | 27 |
| IC26 | Postcentral Gyrus | 15.3505 | -45 | -30 | 51 |
| IC42 | Insula | 14.1456 | 42 | -18 | 12 |
| IC44 | Postcentral Gyrus | 19.4895 | 6 | -45 | 78 |
| Dorsal attention network (DAN) | | | | | |
| IC11 | Inferior Parietal Lobule | 18.0865 | -36 | -51 | 48 |
| IC13 | Superior Temporal Gyrus | 12.7513 | 48 | 6 | -15 |
| IC14 | Middle Temporal Gyrus | 13.8805 | 51 | -57 | 9 |
| IC25 | Precentral Gyrus | 13.6259 | 39 | -48 | 63 |
| IC27 | Superior Parietal Lobule | 15.2087 | -15 | -69 | 66 |
| IC35 | Inferior Parietal Lobule | 20.3882 | 36 | -63 | 51 |
| IC43 | Precuneus | 17.7776 | 9 | -78 | 57 |
| Ventral attention network (VAN) | | | | | |
| IC17 | Medial Frontal Gyrus | 17.9806 | 0 | 12 | 42 |
| IC18 | Superior Temporal Gyrus | 11.8108 | -39 | -12 | -9 |
| IC31 | Anterior Cingulate | 15.6286 | 3 | 12 | 39 |
| IC47 | Superior Frontal Gyrus | 17.1111 | 3 | -3 | 75 |
| Limbic network (LIM) | | | | | |
| IC6 | Parahippocampa Gyrus | 14.7391 | -24 | -15 | -27 |
| IC7 | Superior Temporal Gyrus | 15.3194 | 36 | 15 | -30 |
| IC32 | Insula | 20.7662 | -45 | 9 | -12 |
| Frontoparietal network (FPN) | | | | | |
| IC21 | Middle Frontal Gyrus | 16.029 | -27 | 48 | 24 |
| IC28 | Inferior Parietal Lobule | 16.4565 | 60 | -33 | 48 |
| IC30 | Middle Frontal Gyrus | 16.6809 | 45 | 45 | -12 |
| Default mode network (DMN) | | | | | |
| IC1 | Superior Frontal Gyrus | 22.4692 | -6 | 51 | 30 |
| IC3 | Superior Frontal Gyrus | 15.0009 | -3 | 15 | 63 |
| IC9 | Medial Frontal Gyrus | 18.2377 | 3 | 57 | 0 |
| IC12 | Posterior Cingulate | 17.351 | 6 | -60 | 21 |
| IC23 | Superior Temporal Gyrus | 15.1604 | 51 | -9 | -6 |
| IC37 | Superior Frontal Gyrus | 20.1617 | -24 | 21 | 57 |
| IC39 | Medial Frontal Gyrus | 14.8433 | 6 | 51 | 33 |
| IC41 | Anterior Cingulate | 26.617 | 15 | 30 | -9 |
| IC48 | Precuneus | 20.1341 | 0 | -57 | 36 |
| IC49 | Superior Frontal Gyrus | 16.751 | 0 | 60 | 30 |
| IC50 | Inferior Parietal Lobule | 19.4452 | 57 | -54 | 39 |
| Subcortical network (SCN) | | | | | |
| IC10 | Putamen | 19.0792 | -18 | 3 | 18 |
| IC19 | Thalamus | 16.5349 | -15 | -33 | 6 |
| Cerebellar network (CBN) | | | | | |
| IC2 | Cerebellum Posterior Lobe |  | 15 | -72 | -27 |
| IC38 | Cerebellum Posterior Lobe |  | 9 | -87 | -18 |

**Supplementary Table 2.** Differences in network switching rates between stroke patients and HCs compared using different parameters.

|  | 100 ICs in ICA | Window size of 50 TRs with step length of 1TR | γ = 0.9, ω = 0.5 | γ = 0.9, ω = 1 | γ = 1, ω = 0.5 | γ = 1, ω = 0.75 | γ = 1, ω = 1 | γ = 1.1, ω = 0.5 | γ = 1.1, ω = 0.75 | γ = 1.1, ω = 1 |
| --- | --- | --- | --- | --- | --- | --- | --- | --- | --- | --- |
| Modularity of multilayer network | 0.793 | 0.889 | 0.935 | 0.925 | 0.987 | 0.926 | 0.966 | 0.983 | 0.959 | 0.975 |
| Switching rate of global network | **0.021** | 0.152 | **0.032** | **0.007** | 0.103 | **0.026** | 0.128 | 0.135 | 0.134 | 0.105 |
| Switching rate of subnetwork |  |  |  |  |  |  |  |  |  |  |
| VIS | **0.040** | 0.177 | 0.112 | 0.098 | 0.167 | 0.188 | 0.299 | 0.202 | 0.444 | 0.349 |
| SMN | 0.065 | 0.340 | 0.206 | 0.377 | 0.472 | 0.205 | 0.904 | 0.369 | 0.493 | 0.533 |
| DAN | 0.115 | 0.298 | 0.092 | 0.114 | 0.196 | 0.098 | 0.396 | 0.158 | 0.262 | 0.196 |
| VAN | **0.016** | 0.420 | 0.177 | 0.326 | 0.401 | 0.277 | 0.695 | 0.551 | 0.754 | 0.635 |
| LIM | 0.459 | 0.702 | 0.721 | 0.461 | 0.791 | 0.523 | 0.820 | 0.883 | 0.726 | 0.740 |
| FPN | **0.021** | **0.033** | **0.003** | **0.005** | **0.011** | **0.009** | **0.025** | 0.077 | **0.012** | **0.049** |
| DMN | **0.002** | **0.046** | **0.004** | **< 0.001** | **0.011** | **0.001** | **0.006** | **0.035** | **0.008** | **0.006** |
| SCN | 0.183 | 0.902 | 0.302 | 0.684 | 0.598 | 0.526 | 0.732 | 0.901 | 0.539 | 0.697 |
| CBN | 0.215 | 0.714 | 0.081 | **0.040** | 0.449 | 0.050 | 0.340 | 0.263 | 0.261 | 0.355 |
